# Effect of Warm-Up Exercise on Functional Regulation of Motor Unit Activation during Isometric Torque Production

**DOI:** 10.5114/jhk/185157

**Published:** 2024-04-25

**Authors:** Jiseop Lee, Dawon Park, Joo-Young Lee, Jaebum Park

**Affiliations:** 1 Department of Physical Education, Seoul National University, Seoul, South Korea.; 2 Division of Geriatrics, Department of Internal Medicine, Yonsei University College of Medicine, Seoul, South Korea.; 3 Advanced Institute of Convergence Science, Seoul National University, Seoul, South Korea.; 4 Department of Fashion and Textiles, Seoul National University, Seoul, South Korea.; 5 Research Institute for Human Ecology, Seoul National University, Seoul, South Korea.; 6 Institute for Sports Science, Seoul National University, Seoul, South Korea.; 7 Department of AI-Integrated Education, Seoul National University, Seoul, South Korea.

**Keywords:** body temperature, firing rate of motor unit, recruitment threshold of motor unit, voluntary muscle force production, neuromuscular mechanism, decomposition EMG

## Abstract

In this study, we tested several hypotheses related to changes in motor unit activation patterns after warm-up exercise. Fifteen healthy young men participated in the experiment and the main task was to produce voluntary torque through the elbow joint under the isometric condition. The experimental conditions consisted of two directions of torque, including flexion and extension, at two joint angles, 10° and 90°. Participants were asked to increase the joint torque to the maximal level at a rate of 10% of the maximum voluntary torque. The warm-up protocol followed the ACSM guidelines, which increased body temperature by approximately 1.5°C. Decomposition electromyography electrodes, capable of extracting multiple motor unit action potentials from surface signals, were placed on the biceps and triceps brachii muscles, and joint torque was measured on the dynamometer. The mean firing rate and the recruitment threshold of the decomposed motor units were quantified. In addition, a single motor unit activity from the spike train was quantified for each of five selected motor units. The magnitude of joint torque increased with the warm-up exercise for all the experimental conditions. The results of the motor unit analyses showed a positive and beneficial effect of the warm-up exercise, with an increase in both the mean firing rate and the recruitment threshold by about 56% and 33%, respectively, particularly in the agonist muscle. Power spectral density in the gamma band, which is thought to be the dominant voluntary activity, was also increased by the warm-up exercise only in the high threshold motor units.

## Introduction

As a well-known preparatory physical activity, warm-up exercise has a temporary and brief effect on the muscles as compared to the effects of the long-term treatment such as strength training. The effects of the warm-up are mainly related to their force production as the primary function of the muscle (Bishop, 2003; Güllich and Schmidtbleicher, 1996). The functional importance of the muscle is primarily to convert chemical energy into mechanical work and heat (Hill, 1949). Although the Hill’s muscle model implements the mechanical terms with spring analogy to describe the relationship between the output force by the muscle and its length, velocity, and acceleration, it was discovered that the heat in the active muscles was proportional to the work performed by the muscle (Hill, 1964) like a mechanical motor. Therefore, it is highly probable that the changes in temperature directly in the muscles or indirectly in the whole body would influence the activation patterns of the involved muscles.

It is a well established fact that warm-up exercise results in a significant increase in the force-generating capacity of skeletal muscle (Bergh and Ekblom, 1979). In particular, the basic premise of the warm-up effect is addressed by the temperature-related mechanism (Asmussen and Bøje, 1945; Bishop, 2003), which primarily addresses peripheral changes in muscle physiology, such as muscle metabolism along with the blood flow (Gray et al., 2011), and muscle fiber conduction velocity (Pearce et al., 2012), by increasing muscle temperature. Due to the relatively short duration of treatment, warm-up exercise is not expected to induce dramatic changes in muscle functions in terms of physical quantities such as muscle mass, size, and density. Therefore, it is most likely that the functional changes following the warm-up are assumed to be the combined effects of peripheral and central causes, although estimating the relative contribution of the peripheral and central factors is rather difficult. Nevertheless, a set of variables that encompasses both the peripheral and central factors are necessarily considered to provide a better understanding of the effect of warm-up exercise, which was associated with the rationale of the experiment in this study.

Muscle force is the product of two separable functions, the number and firing frequency of motor units (MUs) recruited within a particular muscle, such that *Force* = Σ*f_1_*(F_MU_)•*f_2_*(ǿ_MU_), where *f_1_* and *f_2_* are the functions of the number of motor units and the frequency, i.e., firing rate, respectively, in relation to muscle force (Broman et al., 1985; Čoh et al., 2010; Heckman and Enoka, 2012). The motor unit is the smallest functional “unit” of muscle activation, consisting of the alpha motoneurons in the spinal cord and a group of muscle fibers. The central nervous system (CNS) mechanism for the voluntary (or in some cases involuntary, e.g., reflex response) changes in muscle force is impacted by the regulation of the two functions mentioned above, i.e., the numbers and frequencies of motor unit (MU) action potentials. There is no doubt about the beneficial effects of the warm-up as a preparatory activity, but little attention has been paid to the investigation of the CNS mechanism as to the regulation of MU activations.

In experiments on muscle response to the treatment, i.e., the warm-up in this study, considerable research attention has been paid to changes in muscle activation and the corresponding mechanics, e.g., outcome force or torque. Surface electromyography (sEMG) has become a popular technological method for measuring muscle activity due to its convenience of use and less movement restriction during measurement compared to intramuscular EMG (Türker, 1993). However, the intramuscular EMG has an inherent methodology for capturing a limited number of individual motor units and their properties, such as firing frequencies. Recently, advanced techniques and analytical algorithms for identifying a set of motor unit action potentials (MUAPs) from interference of multichannel EMG recordings, the so-called ‘decomposition EMG (dEMG)’, have been introduced (De Luca and Nawab, 2011; Nawab et al., 2010). The validity and compatibility of dEMG with intramuscular EMG have been confirmed, especially under the condition of isometric muscle contraction (Hu et al., 2013, 2014).

It is very likely that both the number and the firing frequency of the recruited MUs would be increased by the warm-up exercise. However, a question “how many MUs and at what firing frequencies would the CNS recruit to cause the enhanced muscle force production?” has remained unanswered. The recruitment order among a pool of MUs is well constrained by the size principle (Henneman, 1957; Henneman et al., 1965). However, the size principle is not a key constraint in identifying and understanding the altered muscle function after the warm-up mechanism due to the lack of knowledge of the frequency band of individual MUAPs.

Therefore, the current study explored the neuromuscular mechanism of the warm-up exercise by quantifying the properties of the recruited motor units, including firing frequencies and recruitment thresholds during the isometric torque production at the elbow joint. We formulated three hypotheses: 1) the magnitude of joint torque would be larger after the warm-up exercise, 2) the number and average firing frequencies of the recruited motor units on agonist muscle would increase with the warm-up exercise, while these changes would not be shown in antagonist muscle, and 3) the changes in the firing frequencies would be larger as compared to the changes in the number of MUs with the warm-up exercise.

## Methods

### Participants

Eighteen healthy male participants (age: 32 ± 4.3 years, biceps fat thickness: 1.2 ± 0.3 mm, MMT grade 5, BMI: 18.5 to 24.9) with no medical history of musculoskeletal injuries, neurological disorders, or uncorrected visual acuity deficits and dysfunction, voluntarily participated in the experiment. A priori sample size estimation using G*Power (Faul et al., 2017) for an effect size (f) greater than 0.25, i.e., a medium effect, with at least 80% power (β = 0.8) and a type-I error rate of α = 0.05 given the current experimental protocols, suggested a minimum of 16 participants. Prior to the experiment, participants were fully informed of the procedures and potential risks of the study. They provided written informed consent, which was approved by the Institutional Review Board of the Seoul National University (IRB No. 2004/002-016; approval date: 13 April 2020). The original signed consent form was kept in the experimental records, and a copy was given to each participant.

### Measures

**Figure 1:**
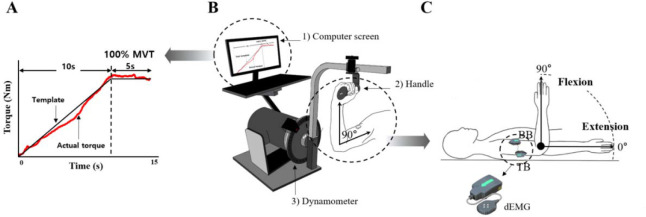
Experimental setting. MVT template on the computer screen (A), experimental device :1) control platform and display, 2) elbow handle, 3) dynamometer (B), and the definition for the range of movement with an elbow joint and tested muscles, biceps brachii (BB) and triceps brachii (TB) muscles with dEMG (decomposition EMG) (C).

A Human Norm dynamometer (Computer Sports Medicine Inc., CSMI, USA) was used to measure joint torque during the experiment. The computer screen was connected to the dynamometer, which provided the torque template (Figure 1A) and the torque exerted by the participant in real time (Figure 1B). A Delsys Decomposition EMG system was used to identify multiple motor units using a specialized surface EMG electrode (Trigno Galileo sensor, Delsys Inc.) (Figure 1C). The dEMG electrodes were placed on the biceps brachii (BB) and triceps brachii (TB) muscles according to the recommendations of the SENIAM guidelines. The EMG signals were sampled at 2000 Hz and band-pass filtered at 20–450 Hz. A digital thermometer (Omron Healthcare Inc.) and a non-contact infrared thermometer (Fluke Corp Inc) were utilized to measure skin surface temperature at three sites, including the forehead, armpits, and the upper arm, which confirmed a significant correlation with muscle temperature (De Ruiter et al., 1999).

### Design and Procedures

Each participant visited the laboratory twice. On the first visit, participants had a practice session to become familiar with the experimental task and the devices. On the second visit, firstly, the skin over the biceps and triceps muscles was cleansed with rubbing alcohol. The dEMG electrode heads were placed near the centroid of the muscle to detect the maximum amount of the EMG activity guideline (Hermens et al., 2000). The task comprised 1) auxiliary maximal voluntary torque (MVT) production and 2) ramp torque production as the main task in the elbow flexion and extension efforts under two joint angle conditions, including 10° and 90°. In addition, the two tasks were repeated before and after the warm-up exercise. Three of the 18 participants did not complete all the experimental conditions; therefore, the data from these participants were excluded from the analysis.

#### Joint Torque Measurement

For the measurement of joint torque, participants were placed in a supine position, and the frame length of the dynamometer was adjusted to account for individual variations in the arm anatomy (Figure 1B). They were instructed to rotate the elbow joint along an axis aligned with the lateral epicondyle of the elbow joint. Once the desired joint angle was set, the frame was locked to ensure a static (i.e., isometric) condition. The chest, the abdomen, the pelvis, and lower extremities were stabilized with a strap to isolate the elbow joint. The elbow joint angle was defined such that a fully extended elbow joint corresponded to 0° (Figure 1C). For the MVT task, participants were required to reach the maximum level of joint torque within 5 s, and the average value across three attempts for each condition was used to scale the torque values of the main task. The main task was the ramp torque production from 0 to 100% of the MVT value with 10% MVT/s as the slope of the ramp, followed by the phase for maintaining 100% of the MVT for approximately 3 s. The torque template and the real-time torque produced by the participant were displayed on the screen, and participants were instructed to match the produced torque to the template as accurately as possible. Two trials were given for each condition, and the order of the direction (two levels: flexion and extension) and the angle (two levels: 10° and 90°) was randomized. The rest interval between trials was 3 min, and all these trials were performed in the same way before and after the warm-up exercise.

#### Warm-Up Exercise Protocol

**Figure 2:**
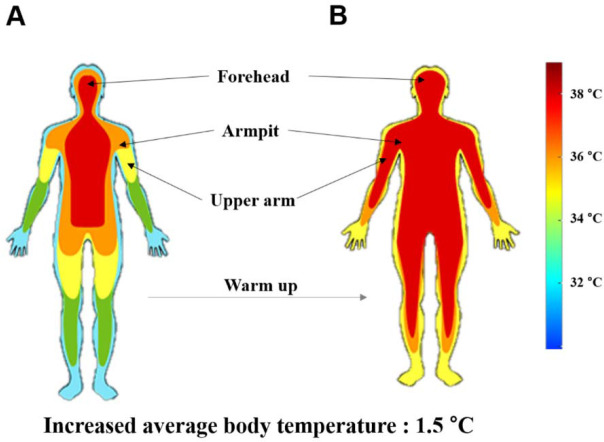
Temperature measuring before the warm-up (A) and after the warm-up (B).

The warm-up exercise lasted approximately 20 min. The obvious aim of the warm-up was to elevate the body temperature, thus the criterion for confirming completion of the warm-up was an increase in the temperature by about 1.5°C at the three measurement sites (Racinais and Oksa, 2010) (Figure 2). The protocol consisted of three separate steps. Step 1 included aerobic exercise, which was 10-min treadmill jogging according to the American College of Sports Medicine (ACSM) guidelines (Swain et al., 2007). Intensity was adjusted between 30 and 60% of heart rate recovery using a heart rate monitor (Pescatello et al., 2014). Step 2 comprised foam rolling on the biceps, the triceps, the back and shoulders with a rigid roller, 30–60 s per section (Wiewelhove et al., 2019). Step 3 consisted of a 5-min active warm-up of the upper body, including 30 s of each of eight Thera-Band exercises (McCrary et al., 2015). Movement frequency in steps 2 and 3 was precisely controlled using a metronome for specific times and repetitions.

#### Data Analysis

Component MU action potential (MUAP) waveforms and firing times were extracted from multichannel surface EMG signals using decomposition algorithms designed to track the firing behavior of individual MUs (NeuroMap software; Delsys Inc.; De Luca et al., 2015). Since the torque profile of the current task was a slow, ramp-like torque production rather than a dynamic torque production (i.e., sinusoidal), a Hann window filter with duration of 1 s was applied to transform the inherently discrete sequences of MUAPs into the ramp-like torque profile. This method effectively combined the effects of two functions, the number of MU recruits and the frequency modulation (De Luca et al., 2006). This result was further validated using the decompose-synthesis-decompose-compare method (Nawab et al., 2010). The mean value of the estimation accuracy of the firing instances tested on a set of 120 contractions was 92% on average. Three variables were extracted: 1) the recruitment threshold, 2) the firing rate at recruitment, and 3) the peak firing rate. The recruitment threshold was calculated as the torque level at which the motor unit started to fire. The firing rate at recruitment was estimated as the inverse of the mean of the first three inter-pulse intervals, and the peak firing rate was calculated as the mean of the mean firing rate trajectory during the duration of constant MVT torque. Linear regressions were performed on firing rates at recruitment and peak firing rates versus the recruitment threshold. The algorithms used artificial intelligence techniques to separate superimposed action potentials and assign them to a single train belonging to a specific motor unit. This technique typically identifies all the firings of 20–30 MUAP trains per contraction.

Upon the completion of the decomposition process, we selected five motor units among a set of decomposed MUs (Park et al., 2016, 2019) for both agonist and antagonist muscles, separately. The criteria and order of the identification were 1) the first recruited motor unit (MU_1_) represented the smallest motor unit in the pool; 2) the last recruited motor unit (MU_5_) represented the largest motor unit in the pool; 3) MU_3_ was the MU in the middle between MU_1_ and MU_5_ as the averaged firing behavior; 4) MU_2_ was identified in the between MU_1_ and MU_3_ as the representative of the lower-threshold MU; and 5) MU_4_ was the MU in the between MU_3_ and MU_5_ as the example of the higher-threshold MU. This approach was assumed to reduce the influence of the discharge rate and maximize the reliability of the comparisons (Gallego et al., 2015; Negro and Farina, 2011).

We calculated the properties of each of five MUs and multi-MUs encompassing all five MUs, separately. For the single-MU analysis, the data of inter-spike intervals of each MU (e.g., MU_1_ to MU_5_) was first acquired, followed by the Fourier transform. The power spectral densities of four predefined frequency bands were estimated and integrated: 1) δ-band: 0–4 Hz; 2) α-band: 4–10 Hz; 3) β-band: 10–35 Hz; and 4) γ-band: 35–60 Hz (Brown et al., 1998; De Luca and Erim, 2002).

For the multi-MU analysis, mean and standard deviations (SD) of the sum of discharge rates across all MU_1_ to MU_5_ were calculated for each condition and participant. The mean discharge rate was quantified as the average of the inter-spike interval, which reflected the time between two consecutive spikes within the time windows of the analysis.

### Statistical Analysis

Descriptive statistics and parametric methods were employed, and the data were presented as means and standard errors. Repeated measures (RM) ANOVAs were performed with the factors *Warm-Up* (two levels: before and after), *Angle* (two levels: 10° and 90°), and *Direction* (two levels: flexion and extension) on a set of outcome variables described in the Methods.

The RM ANOVA on the values of the maximal voluntary torque (MVT), the mean firing rate (MRF), and the recruitment threshold (RT) with the factors *Warm-Up* × *Angle × Direction* was performed to test hypotheses 1 & 3. The RM ANOVA on the mean and SD of the discharge rate from the multi-MU data with the factors *Warm-Up* × *Angle × Direction* was performed to test hypothesis 2. For the single-MU data, three-way RM ANOVAs with the factors *Warm-Up* × *Angle × Direction* were performed on the integral values of the power spectral density in each of the four frequency-band (α-, β-, γ-, δ-band) separately to provide an additional support for hypothesis 2.

In addition, regression analyses were performed to identify the patterns of the significant changes between the MRF and RT on agonist and antagonist muscles, separately. Post hoc estimates of effect size are presented as eta-squared (ƞ^2^) for all comparisons, and the level of significance for all statistical analyses was set at *p* < 0.05. These analytical procedures were performed using IBM SPSS version 24.0 (IBM, Armonk, NY, USA).

## Results

### Changes in the Magnitude of Joint Torque

**Figure 3:**
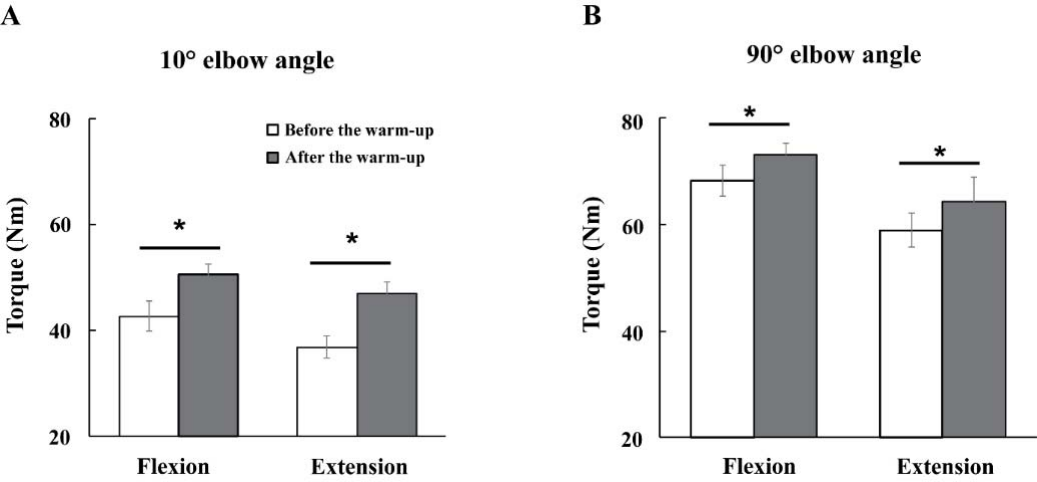
Average maximal voluntary torque (MVT) (Nm) before (white bar) and after (gray bar) warm-up exercise. Elbow joint angle 10° (A), and elbow joint angle 90° (B) (*, *p* < 0.05). Bars represent the mean values with standard error (SE) across participants.

The magnitudes of maximal voluntary torque (MVT) were increased with the warm-up exercise by about 13% across all experimental conditions (Figure 3). Further, the MVTs were larger under the 90° compared to the 10° condition for all direction conditions. Likely, the MVTs during flexion were larger than during extension efforts. These findings were supported by three-way repeated measures ANOVA with factor *Warm-Up* (two levels: before and after), *Angle* (two levels: 10° and 90°), and *Direction* (two levels: flexion and extension), which showed significant main effects of *Warm-Up* (F_[1,14]_ = 31.89, *p* < 0.001, η^2^ = 0.045), *Direction* (F_[1,14]_ = 11.80, *p* < 0.004, η^2^ = 0.048), and *Angle* (F_[1,14]_ = 185.89, *p* < 0.0001, η^2^ = 0.464) with no factor interactions.

### Changes in the Number of Motor Units

**Figure 4:**
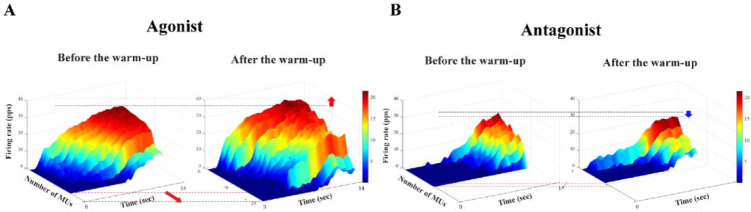
3D plot axis. The data are from single trials performed by a representative participant before and after the warm-up intervention from agonist (A) and antagonist (B) muscles. Motor units decomposed from the surface EMG signal obtained for the biceps brachii (BB) at the 90° elbow angle.

As shown in Table 1, the average number of MUs used in the data analyses was significantly increased by approximately 22% after the warm-up when the muscles were acting as agonist in torque production. However, there was no statistical difference in the number of MUs in antagonist muscles. The results of the three-way repeated measures ANOVA with factor *Warm-Up* (two levels: before and after), *Angle* (two levels: 10° and 90°), and *Direction* (two levels: flexion and extension), showed a significant main effect of *Warm-Up* (F_[1,14]_ = 22.22, *p* < 0.0001, η^2^ = 0.083) with no factor interactions (Figure 4).

**Table 1:** Number of decomposed motor units and the percentage of decomposition accuracy.

Warm-up Direction Elbow angle (°)		Before the warm-up				After the warm-up			
		Flexion		Extension		Flexion		Extension	
		10°	90°	10°	90°	10°	90°	10°	90°
Number of MUs /	Agonist	16 / 92%	15 / 93%	18 / 93%	17 / 91%	21 / 92%	18 / 92%	21 / 92%	20 / 92%
Decomposition accuracy in %	Antagonist	10 / 91%	9 / 92%	9 / 92%	9 / 92%	9 / 93%	11 / 91%	10 / 92%	7 / 91%

### Relationship between the Mean Firing Rate (MFR) and the Recruitment Threshold (RT)

**Figure 5:**
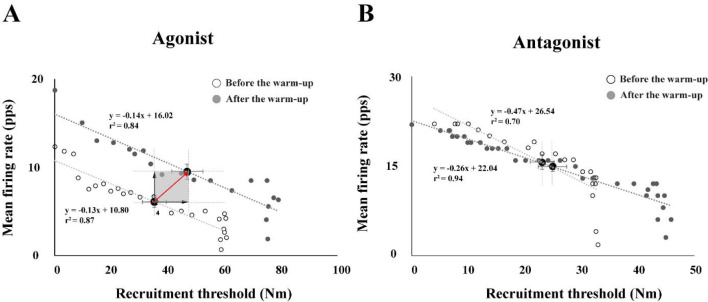
Linear regression between the mean firing rate (MFR) and the recruitment threshold (RT) separately for agonist (A) and antagonist (B) muscles. The data are from single trials performed by a representative participant before and after the warm-up. The white dots represent the individual motor units before the warm-up, and the dark gray dots represent the individual motor units after the warm-up. Moreover, black dots represent the average values and standard error (SE) under the 90° elbow flexion condition. pps: pulse per second.

We analyzed the relationship between the mean firing rate (MFR) and the MU recruitment thresholds (RT) of MUs observed during the MVT. The RT of each MU was recorded as the force level achieved, measured as a percentage of the MVT, at the point where the first MU firing occurred. Figure 5 shows the linear regression illustrating the relationship between the MFR and the RT from the data of a representative participant. After the warm-up protocol, linear slopes were decreased with larger values of the y-intercepts, with a significant effect of the warm-up (Figure 5A). The three-way repeated-measures ANOVAs with factors *Warm-Up* (two levels: before and after), *Angle* (two levels: 10° and 90°), and *Direction* (two levels: flexion and extension) on the MVT torque, showed significant main effects of *Warm-Up* (F_[1,14]_ = 5.41, *p* < 0.03, η^2^ = 0.024) and *Direction* (F_[1,14]_ = 69.63, *p* < 0.0001, η^2^ = 0.306) with no factor interactions.

### Motor Unit Activity with Multi-MUs and Single-MU

Due to the large inter-subjects’ variability in the outcomes of the antagonist muscle analyses, no significant effects were observed in the antagonist muscle variables for both the single and multi-MU analyses. Therefore, the following results were extracted from the agonist muscle variables only.

**Figure 6:**
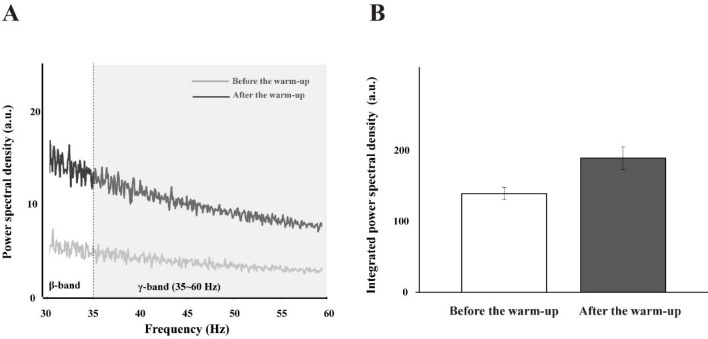
The power spectral density is illustrated before the warm-up (the light gray line) and after the warm-up (the dark gray line) in γ-band (35–60 Hz) (A), and the integrated power spectral density is also presented with two corresponding bars: before the warm-up (the white bar) and after the warm-up (the gray bar) (B). These bars represent the mean values of their respective standard ± SE aggregated across all participants.

For the multi-MU data, the mean discharge rate across five selected MUs increased with the warm-up exercise by about 15% across all the experimental conditions (Table 2), which was confirmed by a significant main effect of *Warm-Up* (F_[1,14]_ = 13.23, *p* < 0.003, η^2^= 0.071), with no other main and interaction effects. On the contrary, the SDs of discharge were similar across all the experimental conditions, which showed no significant outcome. For the single-MU data, the effect of warm-up exercise was dominantly observed in the integral power on γ-band (35–60 Hz) of MU_3_–MU_5_ by showing an about 25% increase after the warm-up exercise. These results were supported by the significant main effects of *Warm-Up* (MU_3_: F_[1,14]_ = 12.38, *p* < 0.003, η^2^ = 0.053; MU_4_: F_[1,14]_ = 11.731, *p* < 0.004, η^2^ = 0.074.; MU_5_: F_[1,14]_ = 12.55, *p* < 0.003, η^2^ = 0.054). The integral power of MU_1_ and MU_2_ showed no significant difference in all the conditions (Figure 6).

**Table 2:** Means and standard error (SE) of the discharge rate across five selected MUs.

Warm-up Direction Elbow angle (°)	Before the warm-up				After the warm-up			
	Flexion		Extension		Flexion		Extension	
	10°	90°	10°	90°	10°	90°	10°	90°
Mean discharge rate (pps)	79 ± 3.2	79± 4.8	100 ± 7.0	84 ± 5.4	100 ± 8.7	88 ± 5.8	102 ± 7.7	100 ± 7.1
SD of the discharge rate (pps)	90 ± 5.6	112 ± 8.4	86 ± 5.0	79 ± 4.7	92 ± 4.9	88 ± 6.3	96 ± 7.9	89 ± 8.6

Values are means ± SE across participants; pps: pulse per second.

## Discussion

In this study, we aimed to examine the effects of the warm-up exercise at the level of the motor unit, which is the smallest functional unit in the neuromuscular structure. The results confirmed most of our hypotheses by showing increases in force (or torque) production capability and an increased number of MUs and their firing rate after the warm-up exercise. These observations lead to a series of possible implications regarding the beneficial effect of the warm-up exercise on muscle force production.

### Changes in Firing Patterns of Agonist Muscle with the Warm-Up Exercise

A direct effect of the warm-up exercise is to increase body temperature, which is presumably related to muscle temperature. In the current experiment, we measured the skin temperature as the index of the warm-up completion rather than a direct measure of muscle temperature due to the complexity of the direct measure and the time constraints of the protocol (i.e., the washout of the treatment effect). While there may be a discrepancy between skin and intramuscular temperature, previous research has found an almost linear relationship between the two measures (De Ruiter et al., 1999). Of course, it is obvious that the current measure of the skin temperature was not the measured index, but the estimated one, which by itself has the limitation such as the error component during the estimation procedure. Nevertheless, the current measurement of the temperature by the non-contact method would be an acceptable index for the process and completion of the warm-up exercise.

The main physiological process of the warm-up exercise is represented by the temperature-related mechanism, which accounts for the metabolic changes such as the blood flow to the muscle due to heat (Asmussen and Bøje, 1945; Bishop, 2003; Gray et al., 2011). Combining the existing knowledge of physiological changes with the current finding of the warm-up effect, the temperature on the muscle has a definite and positive effect on the functional outcomes by the two mechanisms in the MU action potential. The order of recruitment of different types of MUs is well constrained by the size principle (Henneman, 1957; Henneman et al., 1965). From the perspective of the mechanism of MU activation, muscle force is considered a variable that accompanies the activation properties of a set of MUs. The size principle has confirmed the supposition that the order of the MU recruitment depends on their size. The size of MUs is associated with the patterns of twitch and tonic contraction, i.e., the firing rate (Heckman and Enoka, 2012). For each group of MUs, there should be a certain range of twitch and tonic response to some extent, which means that a set of MUs may change their contribution to the net muscle force by changing the firing rate of the action potentials elicited by the corresponding alpha motoneurons. Also, the previous experimental finding of a less significant effect of the changes in the blood flow as metabolic supply on the firing rate of MUs (Murphy et al., 2019) implies that changes in the firing rate after the warm-up exercise may be partly the consequence of the central process rather than the product of changes in the peripheral components. In turn, the role of the controller, the CNS, would focus on the regulation of two functions, the number and the firing rate, of MUs to achieve the goal of the performance. Since the two functional options in the MU action potential would be considered the circumstance of the numerous unknowns, such as the firing rate of individual MUs, with a single constraint by the size principle, i.e., an indeterminate form of the equation, the combinations of these two variables could be the choice of the controller and certain strategies considering the warm-up exercise.

The current results showed a parallel shift of the linear line (i.e., linear regression lines representing “*before*” and “*after*” the warm-up exercise in Figure 5) through the mean firing rate and the recruitment threshold with the extended range of the recruitment threshold (i.e., the recruitment of high-threshold MUs after the warm-up exercise). Seemingly, the firing rate of the particular MU increased with the warm-up exercise showing the parallel scaling of the firing rate with respect to the similar recruitment threshold. In other words, the central output to motor neurons would contribute to the achievement of higher force output. However, the decomposition algorithms used in the current study could not identify whether the MUs with the same recruitment threshold under two conditions were the same MUs or not. The findings of De Luca and Hostage (2010), however, showed that the firing rate of a given MU increased with the force demand, while the recruitment threshold range was extended with the increased number of recruited MUs. Therefore, it is very likely that the increased outcome torque after the warm-up exercise was due to the increased firing rate of the MUs. Nevertheless, we must temper this claim until future studies using intramuscular EMG confirm the veracity of the mechanism of the parallel scaling shown in the current results as a direct reflection of the temperature dependence of the firing rate on a particular MU.

### Possible Neuromuscular Mechanism of Warm-Up Exercise

Based on previous studies examining the optimal performance achieved through the warm-up effect, it has been observed that an increase in muscle temperature leads to an increased blood flow, which in turn improves exercise performance. This improvement can be attributed to changes in both viscosity and stiffness within the muscle (Buchthal et al., 1944). In addition, the physiological mechanism underlying these outcomes is thought to involve an increase in electrical activity of the muscles through stimulation of the neuromuscular system (Stewart et al., 2003). However, there remains limited evidence regarding the precise neuromuscular mechanism by which the warm-up influences muscle force regulation. The beneficial effects of the warm-up on the musculoskeletal system have been attributed to central mechanisms, such as increasing muscle strength and power through expanded MU activation (Duchateau et al., 2006).

The current results show a significant increase in power within the gamma frequency band (35–60 Hz) after the warm-up, which is thought to be indicative of voluntary muscle contractions (Brown et al., 1998). The power density within the gamma frequency band was associated with strengthened high-frequency oscillations in muscle activity, which were linked to improved force control (Ulloa, 2022). Recent analytical approaches based on the uncontrolled manifold hypotheses have been extended to the level of MUs, i.e., covariation patterns between the frequencies of a set of MUs (Madarshahian and Latash, 2021). Therefore, it is highly questionable whether and how the patterns of covariation among multiple MUs would be modified by the warm-up exercise, since the observed changes in the current variables would be in part the reflection of the central process.

The main findings of the experiment provide a few insights into the neuromuscular mechanism of the warm-up exercise as a short-term treatment. The warm-up exercise led to an increase in the force production level of the muscles by simultaneously increasing the firing rate and the number of motor units recruited, especially for the high-threshold motor units, i.e., the type II fast-twitch motor units in the agonist muscle. Therefore, the current findings may provide preliminary knowledge about the muscular conditioning of elite athletes from the neuromuscular perspective, such that the warm-up exercise would be more effective where a high level of force is required. Conversely, the warm-up exercise may not be a good preparatory activity in sports where small yet dexterous muscle control is critical, e.g., rifle shooting, archery, etc. Nevertheless, we have to admit some shortcomings of the current experiment, which should be addressed and answered by follow-up studies. In the experiment, both the agonist and antagonist muscles were measured during the task. However, when the muscle acted as an antagonist, the decomposition outcomes showed relatively large variability across conditions and participants as compared to the agonist. As the current motor task required the production of either flexion or extension torque one at the time, this may be the limiting factor for lower antagonist activation. Perhaps, cyclic torque production by dynamically switching between flexion and extension torque would compensate for the current limitation in data quality of the antagonist muscles. Furthermore, the comparison of the warm-up exercise with the long-term treatments (e.g., a few weeks of strength training) would provide in-depth knowledge about the muscle conditioning of elite athletes.
